# Cognitive behavioral therapy for psychosis: a cost-effectiveness study using the EPiSODe model

**DOI:** 10.1192/j.eurpsy.2025.10028

**Published:** 2025-06-30

**Authors:** Stefan R. A. Konings, Maureen Berkhof, Wim Veling, Ellen Visser, Jochen Mierau, Talitha Feenstra, Richard Bruggeman

**Affiliations:** 1 Department of Psychiatry, Interdisciplinary Center Psychopathology and Emotion Regulation (ICPE), University of Groningen, University Medical Center Groningen, Groningen, The Netherlands; 2 Faculty of Science and Engineering, Groningen Research Institute of Pharmacy, University of Groningen, Groningen, The Netherlands; 3 Rob Giel Research Center, University of Groningen, University Medical Center Groningen, University Center of Psychiatry, Groningen, The Netherlands; 4 University of Groningen, University Medical Center Groningen, University Center of Psychiatry, Groningen, The Netherlands; 5 Faculty of Economics and Business, Department of Economics, Econometrics & Finance, University of Groningen, Groningen, The Netherlands; 6 University of Groningen, University Medical Center Groningen, Groningen, The Netherlands; 7 Lifelines, Groningen, The Netherlands; 8 Center for Nutrition, Prevention and Health Services Research, National Institute for Public Health and the Environment (RIVM), Bilthoven, The Netherlands

**Keywords:** cognitive behavioral therapy, cost-effectiveness, discrete event simulation model, psychotic disorders, scenario analysis

## Abstract

**Background:**

Cognitive Behavioural Therapy for psychosis (CBTp) is an effective psychological treatment for Schizophrenia Spectrum and other psychotic Disorders (SSD). Despite guidelines recommending CBTp for all psychotic disorder patients, many SSD patients lack access to the treatment and little is known about its long-term cost-effectiveness. The aim of this study is to evaluate the cost-effectiveness of CBTp for the treatment of psychotic disorders through scenario analysis from a healthcare perspective.

**Methods:**

Increased implementation of CBTp was evaluated using a real-world SSD population (*N* = 12,835) from the northern Netherlands (2010–2019). A patient-level model was used to simulate the long-term effects of rehospitalisation rate. We compared treatment as usual (TAU) with the same TAU plus hypothetical CBTp for all individuals not having received such in TAU, hence patients who received any CBTp sessions prior were excluded (*N* = 2,679). Outcomes considered were quality-adjusted life years gained and total costs of mental healthcare. Additional sensitivity and scenario analyses were performed to evaluate structural and parameter uncertainty.

**Results:**

TAU+CBTp was a cost-effective treatment in 61.2% of the simulations. The simulated net present values for QALY gains were 0.038, and for incremental costs were €492 per patient on average, resulting in an expected incremental cost-effectiveness ratio (ICER) of €12,947.

**Conclusions:**

The evaluation shows that CBTp is likely a cost-effective treatment, although results were uncertain. These findings stress the importance of sufficient availability of CBTp for SSD patients. Making CBTp available for all eligible SSD patients may lead to substantial health gains for the SSD population and cost savings from the healthcare perspective in The Netherlands.

## Introduction

Schizophrenia Spectrum and other psychotic Disorders (SSD) have a relatively low prevalence, yet are highly burdensome from both the patient’s and the societal perspective [1–4]. These disorders severely impair the quality of life (QoL), functioning, and social participation of patients [4–7]. Treating SSD is often challenging, considering that more than half of the patients do not respond adequately to current treatments [8, 9]. The main treatment options for patients with SSD are antipsychotic medication combined with psychological treatment [10, 11].

Cognitive Behavioural Therapy for psychosis (CBTp) is an effective psychological treatment for SSD patients [12–18]. CBTp aims to reappraise the meaning and purpose of hallucinations and delusions to reduce distress and improve coping in daily life [19]. To this end, CBTp focuses on a collaboration between patient and therapist, in which they create a personalized case formulation to achieve the patient’s goals and to increase control over symptoms and problems, improving autonomy and self-esteem [20]. According to the National Institute for Health and Care Excellence (NICE) and various (inter)national guidelines, among which the Dutch guideline [21], CBTp is an essential treatment and should be offered to everyone with a psychotic disorder [21–26]. Specifically, the Dutch care standard for psychosis states that CBT should be offered to all patients experiencing subclinical psychotic symptoms, psychotic symptoms, and affective symptoms [27].

A recent report by the Dutch Association of Behavioural and Cognitive Therapy (VGCt) highlighted that only 20–25% of the patients who should have been offered CBTp were estimated to have access to the treatment [28]. To improve the quality of CBTp in current practice, more psychologists are needed, and psychologists need more specific training [28]. Internationally, clinical practice is also not in line with guideline recommendations [29–32].

Clinical trials and meta-analyses have shown that CBTp improves positive symptoms [16–18, 20, 33], reduces negative symptoms [18, 33, 34], and improves short-term functioning [35] of SSD patients. Less is known about long term health benefits. Recent meta-analyses found no or small significant effects of CBTp on Quality of Life (QoL) for SSD patients [36–38]. Two meta-analyses have shown that CBTp reduces relapse and rehospitalisation rates, although the uncertainty range around the estimates was large. One meta-analysis reported the relative risk (RR) for relapse based on rehospitalisation (RR = 0.70, CI 0.54–0.91 [10]), the other meta-analysis reported the relative risk of rehospitalisation (RR = 0.79, CI 0.60–1.04 [38]). Both meta-analyses had follow-up times of at most 24 months. Underlying trial populations primarily consisted of individuals with schizophrenia and schizoaffective disorder, with occasional inclusion of other psychotic disorders such as delusional disorder and brief psychotic disorder, with positive symptoms sometimes used as inclusion criteria. To get insight into long-term cost-effectiveness, data synthesis using a simulation model is needed.

A systematic review by Jin et al. [39] showed that the majority of cost-effectiveness studies for SSD evaluated antipsychotics and often used low-quality simulation models. Another systematic review, by Shields et al. [40], showed that the cost-effectiveness of CBTp interventions for psychotic disorders was mostly evaluated in terms of improved functioning (improvement on Global Assessment of Functioning: Haddock et al. [41]; additional days of normal functioning: van der Gaag et al. [35]). One study investigated the incremental health benefits in terms of quality-adjusted life years (QALYs), but this was a trial-based study with a total *N* of 77 and a time horizon of 9 months, only evaluating the cost-effectiveness during the intervention period [42]. Since the systematic review by Jin et al., another simulation study investigated the cost-effectiveness of CBTp for Ultra High Risk individuals [43]. Hence, as of yet, no simulation studies have focused on investigating the effects of CBTp on reduced rehospitalisation or relapse rates and taken a long-term perspective on the cost-utility of CBTp.

To demonstrate the need for proper implementation of this intervention in current clinical practice, we aim to show the potential long-term cost utility of CBTp using simulation modelling. A thorough scenario and sensitivity analysis were performed to deal with the substantial uncertainty around the existing evidence for the effectiveness of CBTp on health-related quality of life (HR-QoL) and healthcare use-related outcomes. Based on these analyses, we aim to draw conclusions about the cost-utility of implemented CBTp for SSD patients from the healthcare perspective. To assist readers without a background in health economics, the online supplementary document (Appendix A) includes a glossary of key health economic terms.

## Methods

A patient-level state transition model was used to simulate the long-term effects of CBTp on Specialized Mental Healthcare (SMH) via the relapse rates. Lower relapse probability leads to less cumulative time in states with reduced HR-QoL and therefore more time is spent in better health states, also reducing healthcare needs. This model, the Evaluating Psychosis by Simulating Outcomes for Decision support (EPiSODe) model, has been validated and is more extensively described on our Open Science Foundation page https://osf.io/k56sp/?view_only=5c1753079c44440cb73fc931aed255e5.

Effect sizes and uncertainty ranges for the relapse and rehospitalisation rates, and HR-QoL utility weights corresponding to model states were based on published literature. Further model parameters were estimated from routine care data from SMH in the northern Netherlands over the period 2000 to 2019. The initial 10 years of data were used to create a baseline population, while the following 10 years of data were used for internal validation of the model. Scenarios with and without full implementation of CBTp were compared and sensitivity analyses were performed.

### Study sample

Administrative registry data with basic patient characteristics (age, sex, and diagnosis) and detailed healthcare use (both in- and outpatient care recorded on a daily basis) were available for (*N* = 12,835) SSD patients receiving SMH in the north of the Netherlands. The catchment area consisted of the provinces Groningen, Friesland, and Drenthe, and the four major SMH providers in this area collected the data. All diagnoses were established by qualified psychologists and psychiatrists in a clinical setting, using the DSM-IV criteria, and were available to select SSD patients for the purposes of this study (more information in Appendix B). Unless they actively avoid care, SSD patients will be treated in SMH.

### Model

Healthcare use trajectories were simulated using a patient-level continuous-time state transition model for SSD. This model distinguishes three healthcare use states representing “in-episode,” “out-of-episode,” and death. The “in-episode” state is defined as a period of increased use of specialized mental healthcare, and distinguishes between episodes with inpatient care and episodes with only outpatient care. The “out-of-episode” state is defined as a period of decreased healthcare. Within this framework, “relapse” is defined as the transition from out to in-episode, while “remission” is defined as the reverse. Mortality was modelled as a transition from either the ‘in-episode’ or the ‘out-of-episode’ state to an absorbing ‘death’ state, using parametric distributions estimated with the available data [44]. The time horizon used was 10 years.

### Intervention and comparator

For the purposes of the current study, CBTp was defined as a psychological intervention based on Dutch guidelines [21]. This involved a minimum of 16 sessions of individual therapy provided by a qualified practitioner, with each session assumed to last approximately 1 hour. Patients who have been identified to have received treatment were excluded from the study sample, resulting in a sample of patients who did not receive CBTp. This sample of patients was used in the simulation model.

We compared treatment as usual (TAU) with the same TAU plus hypothetical CBTp for all individuals not having received such in TAU. For TAU, we simulated actual healthcare use and QALYs for the selected patient population and time frame. For TAU+CBTp, we repeated this with increased one-time treatment costs as a result of CBTp and adjusted sojourn times. The adjusted sojourn times were based on the reduced rehospitalisation or relapse rates resulting from the hypothetical CBTp treatment. The differences in simulated costs and QALYs estimated the long-term effect of providing a proper CBTp treatment to all patients.

### Costs

Treatment costs were calculated as the hourly wage rate of the practitioner times the duration of the therapy in hours. The number of therapy sessions was set to 16 with a cost of €108.22 per session (total treatment costs of €1731.52 per patient). By assumption, CBTp did not incur severe adverse effects. Other costs, such as travel costs or additional education costs per patient for medical practitioners (e.g. resulting from a required course or obtaining a qualification) were assumed as negligible for the purposes of the current simulation study. Unit costs were obtained and indexed for 2019 and determined using the Dutch costing manual [45].

At each iteration, the simulation model uses cost equations to assign a level of costs for each patient based on their current model state, modelled sojourn time, and other patient characteristics. Transitions to the episode state lead to an increase in costs, while transitions to the out-of-episode state imply a cost decrease. In this way, individual patients will differ in the level of costs, reflecting the large variation in intensity and type of care provided to Schizophrenia patients. In principle, as the simulated patients are assumed to transition less frequently to the “in-episode” state after receiving CBTp, these patients use less costly SMH.

### Health effects

The effectiveness of CBTp on rehospitalisation rates was estimated as a relative risk, based on two reviews [10, 38]. The duration of this treatment effect was conservatively assumed to be 2 years, which was the maximum follow-up time of the underlying randomized controlled trials (RCTs). In sensitivity analyses, we varied the treatment effect duration from 1 to 10 years.

Long term health benefits were estimated in QALYs gained by keeping track of the total time in episode with outpatient care, the total time in episode with inpatient care and the total time in a stable out of episode state and multiplying each with their respective health related quality of life weight (Table OS1 in the Online Supplement). HR-QoL weights were taken from a review by Zhou J et al. [46]. The utility values estimated by Briggs et al. [47] based on a Time Trade-off (TTO) instrument were chosen to be the most recent and suitable HR-QoL weight estimates for our model states. These estimates have also been used in existing cost-effectiveness studies for antipsychotics [48–50]. In line with existing cost-effectiveness studies for antipsychotics with similar states, the HR-QoL value for the in-episode with outpatient care state was estimated as the average QoL value of the two other (best and worst) states, while the largest observed standard error was used to model uncertainty. Base case estimates from the patient sample were selected as a conservative assumption (see Table OS1 in the Online Supplement).

Model simulations resulted in total QALYs and total costs for the simulated population for each scenario, per year. These were used to calculate net present values, using a discount rate of 3.5% for both costs and QALYs as per the UK guideline [51]. In a scenario analysis, a discount rate of 4% for costs and 1.5% for QALYs was used as per the Dutch guidelines [52].

Finally, the intervention was considered as being cost-effective if the Incremental Cost Effectiveness Ratio (ICER) did not exceed a Willingness To Pay (WTP) threshold of €50,000 [53].

### Sensitivity analyses

One-way sensitivity analyses were used to investigate the importance of the model assumptions concerning treatment effect duration, hourly medical practitioner costs, number of treatment sessions, group therapy (reduced treatment costs p.p.), discount rates, HR-QoL weights, and direct QoL improvements. The results were presented in Tornado diagrams. Furthermore, a probabilistic sensitivity analysis was performed, using 750 outer loops and 250 inner loops (using the method from Oakley et al. [54] to determine these values). Parameters varied in the PSA and their distributions are presented in Table OS2 in the Online Supplement. Constant random seed (Common Random Numbers (CRN)) was used in each pair of simulation comparisons as a variance reduction technique.

## Results

Descriptive statistics for the study population and sample after exclusion criteria are shown in Table 1.

**Table 1.** Overview of the study sample

Variable	Study population *N* = 12,835	CBTp exclusion *N* = 10,156
Male %	7447 (58.0%)	5990 (58.6%)
Age on entry, mean (SD)	38.8 (17.0)	40.4 (17.4)
Follow-up time in years (2010–2019), mean (SD)	8.1 (2.6)	8.0 (2.6)
Number of episodes (p.p.), mean (SD)	3.6 (2.8)	3.8 (2.7)
Episode duration in years (p.e.), mean (SD)	0.2 (0.8)	0.2 (0.9)
Total cost (p.p.), mean (SD)	€33,736	€28,371
Cost per follow-up year, mean (SD)	€4836	€4359
**Primary diagnosis (DSM-IV)**		
−291 (alcohol related), *N* (%)	79 (0.6%)	68 (0.6%)
−292 (drugs related), *N* (%)	567 (4.4%)	444 (4.3%)
−293 (medical condition), *N* (%)	102 (0.8%)	83 (0.8%)
−295 (schizophrenia), *N* (%)	6299 (49.1%)	4912 (48.0%)
−297 (delusional disorder), *N* (%)	1156 (9.0%)	930 (9.1%)
−298 (brief/other psychosis), *N* (%)	5672 (44.2%)	4323 (42.3%)
-Missing (unknown), *N* (%)	682 (5.3%)	620 (6.1%)

*Note:* Individuals may have had multiple primary diagnoses over the course of the study period.

**Figure 1.**
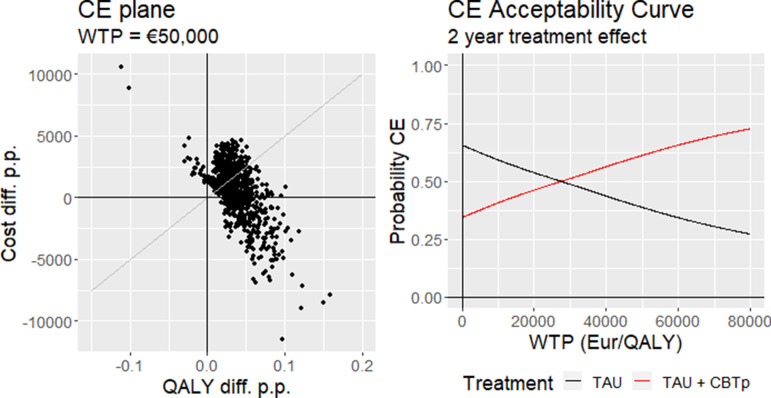
Left: cost-effectiveness plane. Dots represent outer loop draws (parametric uncertainty). WTP line = €50,000 per QALY gained. Right: Cost-effectiveness acceptability curve (CEAC). CE = Cost-effectiveness; WTP = Willingness to pay; QALY = Quality adjusted life year.

The cost-effectiveness plane for the base case scenario with parameter uncertainty is shown in Figure 1. In around a third of the simulations, TAU+CBTp was the dominant treatment with both cost savings and health gains relative to TAU. Assuming a WTP threshold of €50,000 [53], TAU+CBTp was found to be cost-effective in more than 60% of the simulations. The cost-effectiveness acceptability curve is also shown in Figure 1. For a WTP of €80,000, TAU+CBTp would be cost-effective in more than 70% of the simulations.

Table 2 shows the mean results for various scenario analyses. One such analysis is determining the expected additional costs and health benefits for different assumptions on treatment effect duration. The scenario with the shortest treatment duration (1 year) shows a mean simulated 0.031 QALY gain and €2410 in costs per patient for TAU+CBTp compared with TAU. The scenario with the longest treatment duration (10 years) shows a mean simulated 0.061 QALY gain and €1163 in costs per patient for TAU+CBTp compared with TAU.

**Table 2.** Overview of expected cost, QoL differences, and ICER resulting from CBTp treatment, sensitivity analysis assuming different scenarios

Scenario		Expected cost difference	Expected difference in QALYs	ICER
Base case		€492	0.038 years	€12,947
Treatment effect duration	2 year (base)	€492	0.038 years	€12,947
	1 year	€705	0.030 years	€23,500
	3 year	€272	0.043 years	€6,326
	5 year	€98	0.052 years	Dominant
	10 year	€566	0.061 years	Dominant
Rehospitalization risk	0.79 (0.60–1.04) [38]	€492	0.038 years	€12,947
	0.70 (0.54–0.91) [10]	€428	0.051 years	€8,392
QOL weight	Patient sample (base case) [46]	€492	0.038 years	€12,947
	Lay person sample [46]	€492	0.046 years	€10,696
Discount rate	3.5% cost, 3.5% outcome (base case)	€492	0.038 years	€12,947
	4% cost, 1.5% outcome	€506	0.038 years	€13,315

Another analysis shows the impact of using the lower rehospitalisation risks resulting from the meta-analysis by McDonagh et al. [10]. Lower rehospitalisation risks would lead to reduced costs, and a larger health benefit, as shown by this scenario. Using the lay-person sample to determine TTO QoL weights would result in larger health benefits. Finally, we observe that a higher discount rate for costs leads to a lower net present value of cost savings, while the discount rate barely affected the health benefits.

## Discussion

Use of CBTp is likely a cost-effective treatment for SSD patients. Following our base case analysis, TAU+CBTp was the dominant treatment relative to TAU in more than 30% of the simulations, and cost-effective in more than 50% of the simulations. On average, the simulated QALY gain was 0.038, approximately 2 weeks in full health, and the simulated costs were €492 per patient, which were then more than covered by cost reductions as a result of less healthcare use episodes. For patients in the Netherlands this could result in an expected QALY gain of 3157 years for an expected cost of €40.9 million euros. The probability that CBTp is a cost-effective treatment increased with longer treatment effect duration, larger treatment effect, and cheaper treatment.

In line with existing literature, we found that the incremental health benefits for CBTp were relatively small. However, our study also showed that the potential cost savings of proper CBTp implementation could be substantial. To the best of our knowledge, only two studies by Barton et al. [42] and by Jin et al. [43] investigated the cost-effectiveness of CBTp using QALYs as the health benefit outcome. Both studies showed that CBTp could be a cost-effective treatment for psychosis patients. However, Barton et al. did not consider the preventive effect of CBTp on rehospitalisation chance. Moreover, the study by Barton et al. was based on a small trial sample, while we simulated a large population with a long follow-up time. Compared to Barton, our results showed a larger probability of CBTp being a cost-effective treatment. The study by Jin et al. considered people at high risk for psychosis, in contrast to our study, which evaluated CBTp in patients with an existing diagnosis. Their study showed that CBTp could be cost-effective at preventing the onset of the disorder, while we showed that CBTp could be cost-effective for preventing recurrent healthcare use relapses.

Other studies that investigated the cost-effectiveness of CBTp, did not use QALYs, but a variety of short-term outcome measures, which makes it impossible to compare results directly. Studies by Haddock et al., van der Gaag et al., and others [34, 35, 41, 55] have shown that CBTp could improve functioning and (both positive and negative) symptoms in addition to reducing relapse risks, further supporting the benefits of CBTp.

A major strength of the current study was the availability of administrative healthcare use and diagnosis data for a large population of SSD patients in the Northern Netherlands. The patients in our study data were the vast majority of SSD patients in the study catchment area, which was beneficial for the representativeness of our study sample. Furthermore, follow-up was available from 2000 until 2019. As a result, we were able to mitigate the issue of left-censoring by splitting the data in 2010, using the initial 10 years of data to create a baseline population while another 10 years of data were available for internal validation of the model.

By using a state-transition simulation model, we were able to perform a wide range of scenarios and sensitivity analyses. To verify the robustness of our findings, we included uncertainty around regression model coefficients, the rehospitalisation risk, and QoL weights in the PSA. Furthermore, we performed additional analyses with varying assumptions on treatment costs, with parameters extracted from different available meta-analyses, and investigated the impact of alternative QoL weights based on input from a lay-person sample.

A major limitation of simulation modelling using administrative data is the difference between simulated reality and the real world. Reality is inherently more complex than a simulation, and assumptions in the model are generally based on estimations that could be biased or even incorrect. Moreover, estimations performed in small samples or trials could have substantial uncertainty, such as the parameter values used for rehospitalisation risk. Moreover, while the lack of qualified practitioners is the primary barrier to CBTp availability [28], suggesting that missing out on treatment is largely random. However, treatment effect sizes extracted from the literature may be overly optimistic if patients most likely to benefit have been prioritised for treatment and thus excluded from the study [56]. Another limitation of our study is the reliance on the assumption that CBT-p effectiveness is consistent across all subtypes of SSD, primarily because the available trials providing evidence are conducted mostly with schizophrenia patient populations. This assumption was made because the target population includes all patients experiencing subclinical psychotic symptoms, psychotic symptoms, and affective symptoms [27]. While this assumption may not hold for patients with a substance-related psychosis diagnosis, such patients comprised less than 5% of the study population, meaning their exclusion would have minimal impact on our findings.

Since the total number of patients in multiple RCTs included in the meta-analyses was small [38], we recommend that future RCTs consider the inclusion of QoL or rehospitalisation-related outcomes as primary or secondary endpoints in their studies. Another factor affecting the uncertainty around effect sizes is the quality of the CBTp treatment. Duration of the treatment [57], in addition to education level, type, and competence of the medical practitioner, also affect treatment outcomes and effect size uncertainty [58–61]. Furthermore, the primary focus of the treatment has been mentioned as a reason for varying effect sizes per outcome [62, 63]. Additionally, various effect sizes are reported to vary over time after treatment [10].

Another point to consider is the feasibility of adding CBTp to TAU in practice. For instance, when a major reason for the lack of available treatment is a lack of available practitioners, then providing additional CBTp may come with the cost of reducing other beneficial treatments [64]. Such real-world implications are not captured by the model and are beyond the scope of the current study. Enhancing indication practices may be a key aspect of the solution, as findings indicate that CBTp is not cost-effective for a portion of patients, and other findings support the notion that CBTp is not effective for everyone [65–67].

The current study shows the potential of more widespread use of CBTp and hence indicates it might be worthwhile to indeed ensure better availability of practitioners to offer CBTp to those who need it. Since the effectiveness of CBTp varies between patients, perhaps the cost-effectiveness could be further improved by applying a more personalized approach, considering evidence summarized by Newman-Taylor and Bentall hints that relatively small effect sizes mask heterogeneity of treatment outcomes [56].

Moreover, various cost and health differences were not considered in the simulation study. After considering potential societal cost savings such as reduced informal care, and other positive health benefits such as improved functioning or symptoms [68], the treatment could be found to be even more cost-effective relative to our analysis, where we merely consider the potential effect on rehospitalisation. Although psychosocial interventions such as CBTp could sometimes have harmful effects [69], there is a lack of direct evidence that CBTp leads to a significant increase of severe adverse events [38, 70], hence our assumption to omit modelling of such adverse events.

In conclusion, CBTp is likely a cost-effective treatment, with a 61.2% probability of being cost-effective at a WTP of 50.000 euros per QALY and using conservative assumptions about the health benefits of CBTp. These findings show the importance of sufficient availability of CBTp for SSD patients. Proper implementation of and guideline adherence for CBTp could lead to substantial health gains and cost savings for the SSD population in the Netherlands. Further clinical investigation of QoL effects and, in particular, the effect on risk of relapse or rehospitalisation would be required to reinforce these findings.

## Supporting information

10.1192/j.eurpsy.2025.10028.sm001 Konings et al. supplementary material Konings et al. supplementary material

## Data Availability

The data used in this research concerns linked pseudonymised patient level data, suitable for use by researchers, after permission from the members of the IMPROVE consortium. However, due to binding legislation and institutional policy sharing of these data to third parties is not possible.

## Supplementary material

The supplementary material for this article can be found at http://doi.org/10.1192/j.eurpsy.2025.10028.

## Code availability

The code for this project is publicly available on the Open Science Framework (OSF) at https://osf.io/k56sp/?view_only=5c1753079c44440cb73fc931aed255e5.

## References

[1] Christensen MK, Lim CCW, Saha S, Plana-Ripoll O, Cannon D, Presley F, et al. The cost of mental disorders: a systematic review. Epidemiol Psychiatr Sci 2020;29:e161. 10.1017/S204579602000075X. 32807256 PMC7443800

[2] Chong HY, Teoh SL, Wu DB-C, Kotirum S, Chiou C-F, Chaiyakunapruk N. Global economic burden of schizophrenia: a systematic review. Neuropsychiatr Dis Treat. 2016;12(1176-6328 (Print)):357–73. 10.2147/NDT.S96649. 26937191 PMC4762470

[3] Jin H, Mosweu I. The societal cost of schizophrenia: a systematic review. Pharmacoecon. 2017;35(1):25–42. 10.1007/s40273-016-0444-6. 27557994

[4] Charlson FJ, Ferrari AJ, Santomauro DF, Diminic S, Stockings E, Scott JG, et al. Global epidemiology and burden of schizophrenia: findings from the global burden of disease study 2016. Schizophr Bull. 2018;44(6):1195–203. 10.1093/schbul/sby058. 29762765 PMC6192504

[5] Alptekin K, Akvardar Y, Akdede BBK, Dumlu K, Işık D, Pirinçci F, et al. Is quality of life associated with cognitive impairment in schizophrenia? Prog Neuro Psychopharmacol Biol Psychiatr. 2005;29(2):239–44. 10.1016/j.pnpbp.2004.11.006. 15694230

[6] Solanki R, Singh P, Midha A, Chugh K. Schizophrenia: impact on quality of life. Indian J Psychiatry. 2008;50(3):181–6. 10.4103/0019-5545.43632. 19742235 PMC2738356

[7] Sidlova M, Prasko J, Jelenova D, Kovacsova A, Latalova K, Sigmundova Z, Vrbova K. The quality of life of patients suffering from schizophrenia – a comparison with healthy controls. Biomed Pap. 2011;155(2):173–80. 10.5507/bp.2011.010. 21804627

[8] Kennedy JL, Altar CA, Taylor DL, Degtiar I, Hornberger JC. The social and economic burden of treatment-resistant schizophrenia: a systematic literature review. Int Clin Psychopharmacol. 2014;29(2):63–76. 10.1097/YIC.0B013E32836508E6. 23995856

[9] Samara MT, Nikolakopoulou A, Salanti G, Leucht S. How many patients with schizophrenia do not respond to antipsychotic drugs in the short term? An analysis based on individual patient data from randomized controlled trials. Schizophr Bull. 2019;45(3):639–46. 10.1093/SCHBUL/SBY095. 29982701 PMC6483567

[10] McDonagh MS, Dana T, Selph S, Devine EB, Cantor A, Bougatsos C, et al. Treatments for schizophrenia in adults: a systematic review. Compar Effect Rev. 2017;198. 29537779

[11] Muench J, Hamer AM. Adverse effects of antipsychotic medications. Am Fam Physician. 2010;81(5):617–22. 20187598

[12] Turner DT, van der Gaag M, Karyotaki E, Cuijpers P. Psychological interventions for psychosis: a meta-analysis of comparative outcome studies. Am J Psychiatry 2014;171(5):523–38. 10.1176/appi.ajp.2013.13081159. 24525715

[13] Jauhar S, McKenna PJ, Radua J, Fung E, Salvador R, Laws KR. Cognitive–behavioural therapy for the symptoms of schizophrenia: systematic review and meta-analysis with examination of potential bias. Br J Psychiatry 2014;204(1):20–9. 10.1192/bjp.bp.112.116285. 24385461

[14] Pfammatter M, Junghan UM, Brenner HD. Efficacy of Psychological Therapy in Schizophrenia: Conclusions From Meta-analyses. Schizophr Bull, 2006;32(suppl_1):S64–80. 10.1093/schbul/sbl030. 16905634 PMC2632545

[15] Pilling S, Bebbington P, Kuipers E, Garety P, Geddes J, Orbach G, Morgan C. Psychological treatments in schizophrenia: I. Meta-analysis of family intervention and cognitive behaviour therapy. Psychol Med. 2002;32(5):763–82. 10.1017/S0033291702005895. 12171372

[16] Gould RA, Mueser KT, Bolton E, Mays V, Goff D. Cognitive therapy for psychosis in schizophrenia: an effect size analysis. Schizophr Res. 2001;48(2):335–42. 10.1016/S0920-9964(00)00145-6. 11295385

[17] Zimmermann G, Favrod J, Trieu VH, Pomini V. The effect of cognitive behavioral treatment on the positive symptoms of schizophrenia spectrum disorders: a meta-analysis. Schizophr Res. 2005;77(1):1–9. 10.1016/j.schres.2005.02.018. 16005380

[18] Wykes T, Steel C, Everitt B, Tarrier N. Cognitive behavior therapy for schizophrenia: effect sizes, clinical models, and methodological rigor. Schizophr Bull. 2008;34(3):523–37. 10.1093/schbul/sbm114. 17962231 PMC2632426

[19] Birchwood M, Trower P. Cognitive therapy for command hallucinations: not a quasi-neuroleptic. J Contemp Psychother. 2006;36(1):1–7. 10.1007/s10879-005-9000-y.

[20] van der Gaag M, Valmaggia LR, Smit F. The effects of individually tailored formulation-based cognitive behavioural therapy in auditory hallucinations and delusions: a meta-analysis. Schizophr Res. 2014;156(1):30–7. 10.1016/j.schres.2014.03.016. 24731619

[21] Castelein S, Knegtering H, van Meijel B, van der Gaag M. Dutch guideline on schizophrenia 2012: basic care within the areas of psychosocial interventions and nursing care. Tijdschrift voor psychiatrie. 2013;55(9):707–14. 24046249

[22] Health NCCfM. Psychosis and schizophrenia in adults: treatment and management. 2014.

[23] Keshavan MS, Roberts M, Wittmann D. Guidelines for clinical treatment of early course schizophrenia. Curr Psychiatr Rep. 2006;8(4):329–34. 10.1007/s11920-006-0070-7. 16879798

[24] Elisha D, Karny N, Styr BB. [Psychotherapy–outcome studies and guidelines for evidence-based care policy in Israel]. Harefuah. 2011;150(3):269–74, 302. 21574364

[25] Lecomte T, Abidi S, Garcia-Ortega I, Mian I, Jackson K, Jackson K, Norman R. Canadian treatment guidelines on psychosocial treatment of schizophrenia in children and youth. Can J Psychiatry. 2017;62(9):648–55. 10.1177/0706743717720195. 28886670 PMC5593249

[26] Galletly C, Castle D, Dark F, Humberstone V, Jablensky A, Killackey E, et al. Royal Australian and New Zealand College of Psychiatrists clinical practice guidelines for the management of schizophrenia and related disorders. Aust N Z J Psychiatr. 2016;50(5):410–72. 10.1177/0004867416641195. 27106681

[27] Boonstra N, van Gool R, van Duin D. Zorgstandaard Psychotische stoornissen. 2019.

[28] Staring TvdB D, Schuurmans H, van der Vleugel B. Praten naast pillen: krijgt de patiënt met psychose dat wel? 2019.

[29] Haddock G, Eisner E, Boone C, Davies G, Coogan C, Barrowclough C. An investigation of the implementation of NICE-recommended CBT interventions for people with schizophrenia. J Ment Health. 2014;23(4):162–5. 10.3109/09638237.2013.869571. 24433132

[30] Ince P, Haddock G, Tai S. A systematic review of the implementation of recommended psychological interventions for schizophrenia: rates, barriers, and improvement strategies. Psychol Psychother Theory Res Pract. 2016;89(3):324–50. 10.1111/papt.12084. 26537838

[31] Johns L, Jolley S, Garety P, Khondoker M, Fornells-Ambrojo M, Onwumere J, et al. Improving access to psychological therapies for people with severe mental illness (IAPT-SMI): lessons from the South London and Maudsley psychosis demonstration site. Behav Res Ther. 2019;116:104–10. 10.1016/j.brat.2019.03.002. 30877877

[32] Johns L, Isham L, Manser R. Chapter 15 – Cognitive behavioural therapies for psychosis. In: Badcock JC, Paulik G, editors. A clinical introduction to psychosis. Academic Press; 2020, pp. 343–77. 10.1016/B978-0-12-815012-2.00015-8.

[33] Rector NA, Beck AT. Cognitive behavioral therapy for schizophrenia: an empirical review. J Nerv Ment Dis. 2012; 200(10). Reprinted from the *J Nerv Ment Dis*. 2001;189(5);278–87. 10.1097/NMD.0b013e31826dd9af 23034571

[34] Lutgens D, Gariepy G, Malla A. Psychological and psychosocial interventions for negative symptoms in psychosis: systematic review and meta-analysis. Br J Psychiatr. 2017;210(5):324–32. 10.1192/bjp.bp.116.197103. 28302699

[35] van der Gaag M, Stant AD, Wolters KJK, Buskens E, Wiersma D. Cognitive–behavioural therapy for persistent and recurrent psychosis in people with schizophrenia-spectrum disorder: cost-effectiveness analysis. Br J Psychiatr. 2011;198(1):59–65. 10.1192/bjp.bp.109.071522. 21200078

[36] Laws KR, Darlington N, Kondel TK, McKenna PJ, Jauhar S. Cognitive behavioural therapy for schizophrenia – outcomes for functioning, distress and quality of life: a meta-analysis. BMC Psychol. 2018;6(1):32. 10.1186/s40359-018-0243-2. 30016999 PMC6050679

[37] Zheng Y, Xu T, Zhu Y, Li C, Wang J, Livingstone S, Zhang T. Cognitive behavioral therapy for prodromal stage of psychosis—outcomes for transition, functioning, distress, and quality of life: a systematic review and meta-analysis. Schizophr Bull. 2021. 10.1093/schbul/sbab044. PMC8781350 33944949

[38] Jones C, Hacker D, Xia J, Meaden A, Irving CB, Zhao S, et al. Cognitive behavioural therapy plus standard care versus standard care for people with schizophrenia. Cochrane Database Syst Rev. 2018(12). 10.1002/14651858.CD007964.pub2. PMC6517137 30572373

[39] Jin H, Tappenden P, Robinson S, Achilla E, MacCabe JH, Aceituno D, Byford S. A systematic review of economic models across the entire schizophrenia pathway. Pharmaco Econ. 2020;38(6):537–55. 10.1007/s40273-020-00895-6. 32144726

[40] Shields GE, Buck D, Elvidge J, Hayhurst KP, Davies LM. Cost-effectiveness evaluations of psychological therapies for schizophrenia and bipolar disorder: a systematic review. Int J Technol Assess Health Care. 2019;35(4):317–26. 10.1017/S0266462319000448. 31328702 PMC6707812

[41] Haddock G, Barrowclough C, Tarrier N, Moring J, O’Brien R, Schofield N, et al. Cognitive–behavioural therapy and motivational intervention for schizophrenia and substance misuse: 18-month outcomes of a randomised controlled trial. Br J Psychiatry. 2003;183(5):418–26. 10.1192/bjp.183.5.418. 14594917

[42] Barton GR, Hodgekins J, Mugford M, Jones PB, Croudace T, Fowler D. Cognitive behaviour therapy for improving social recovery in psychosis: Cost-effectiveness analysis. Schizophr Res. 2009;112(1):158–63. 10.1016/j.schres.2009.03.041. 19403270

[43] Jin H, Tappenden P, MacCabe JH, Robinson S, Byford S. Evaluation of the cost-effectiveness of services for schizophrenia in the UK across the entire care pathway in a single whole-disease model. JAMA Netw Open. 2020;3(5):e205888. 10.1001/jamanetworkopen.2020.5888. 32459356 PMC7254180

[44] Konings SRA, Mierau JO, Visser E, Bruggeman R, Feenstra TL. Life years lost for users of specialized mental healthcare. Acta Psychiatr Scand. 2023;148(4):338–46. 10.1111/acps.13608. 37697672

[45] Hakkaart-van Roijen L, Van der Linden N, Bouwmans C, Kanters T, Tan SS. Methodologie van kostenonderzoek en referentieprijzen voor economische evaluaties in de gezondheidszorg; 2015. https://www.zorginstituutnederland.nl/publicaties/publicatie/2016/02/29/richtlijn-voor-het-uitvoeren-van-economische-evaluaties-in-de-gezondheidszorg (accessed 30 Sept 2020).

[46] Zhou J, Millier A, François C, Aballéa S, Toumi M. Systematic review of utility values used in the pharmacoeconomic evaluations for schizophrenia: implications on cost-effectiveness results. J Mark Acces Health Policy. 2019;7(1):1648973. 10.1080/20016689.2019.1648973. PMC6713214 31489150

[47] Briggs A, Wild D, Lees M, Reaney M, Dursun S, Parry D, Mukherjee J. Impact of schizophrenia and schizophrenia treatment-related adverse events on quality of life: direct utility elicitation. Health Qual Life Outcome. 2008;6(1):105. 10.1186/1477-7525-6-105. PMC2613374 19040721

[48] Mehnert A, Nicholl D, Pudas H, Martin M, McGuire A. Cost effectiveness of paliperidone palmitate versus risperidone long-acting injectable and olanzapine pamoate for the treatment of patients with schizophrenia in Sweden. J Med Econ 2012;15(5):844–61. 10.3111/13696998.2012.681531. 22458756

[49] Zeidler J, Mahlich J, Greiner W, Heres S. Cost effectiveness of paliperidone palmitate for the treatment of schizophrenia in Germany. Appl Health Econ Health Policy. 2013;11(5):509–21. 10.1007/s40258-013-0050-0. 23975630

[50] Druais S, Doutriaux A, Cognet M, Godet A, Lançon C, Levy P, et al. Cost effectiveness of paliperidone long-acting injectable versus other antipsychotics for the maintenance treatment of schizophrenia in France. Pharmaco Econ. 2016;34(4):363–91. 10.1007/s40273-015-0348-x. PMC4796324 26883132

[51] Attema AE, Brouwer WBF, Claxton K. Discounting in economic evaluations. Pharmaco Econ. 2018;36(7):745–58. 10.1007/s40273-018-0672-z. PMC5999124 29779120

[52] Versteegh M, Knies S, Brouwer W. From good to better: new dutch guidelines for economic evaluations in healthcare. Pharmaco Econ. 2016;34(11):1071–4. 10.1007/s40273-016-0431-y. 27613159

[53] Versteegh MM, Ramos IC, Buyukkaramikli NC, Ansaripour A, Reckers-Droog VT, Brouwer WBF. Severity-adjusted probability of being cost effective. Pharmaco Econ. 2019;37(9):1155–63. 10.1007/s40273-019-00810-8. PMC6830403 31134467

[54] Oakley JE, Brennan A, Tappenden P, Chilcott J. Simulation sample sizes for Monte Carlo partial EVPI calculations. J Health Econ. 2010;29(3):468–77. 10.1016/j.jhealeco.2010.03.006. 20378190

[55] Naeem F, Johal R, McKenna C, Rathod S, Ayub M, Lecomte T, et al. Cognitive behavior therapy for psychosis based guided self-help (CBTp-GSH) delivered by frontline mental health professionals: results of a feasibility study. Schizophr Res. 2016;173(1):69–74. 10.1016/j.schres.2016.03.003. 26971071

[56] Newman-Taylor K, Bentall R. Cognitive behavioural therapy for psychosis: the end of the line or time for a new approach? Psychology and psychotherapy: theory, Res Pract. 2024;97(1):4–18. 10.1111/papt.12498. 37804105

[57] Naeem F, Farooq S, Kingdon D. Cognitive behavioural therapy (brief versus standard duration) for schizophrenia. Cochrane Database Syst Rev.. 2015(10). 10.1002/14651858.CD010646.pub3. PMC8078583 26488686

[58] Muse K, McManus F. A systematic review of methods for assessing competence in cognitive–behavioural therapy. Clin Psychol Rev. 2013;33(3):484–99. 10.1016/j.cpr.2013.01.010. 23454222

[59] Roth AD, Pilling S, Turner J. Therapist training and supervision in clinical trials: implications for clinical practice. Behav Cogn Psychother. 2010;38(3):291–302. 10.1017/S1352465810000068. 20367895

[60] Jolley S, Onwumere J, Bissoli S, Bhayani P, Singh G, Kuipers E, et al. A pilot evaluation of therapist training in cognitive therapy for psychosis: therapy quality and clinical outcomes. Behav Cogn Psychother. 2015;43(4):478–89. 10.1017/S1352465813001100. 24360498

[61] Steel C, Tarrier N, Stahl D, Wykes T. Cognitive behaviour therapy for psychosis: the impact of therapist training and supervision. Psychother Psychosom. 2012;81(3):194–5. 10.1159/000334250. 22433800

[62] Velthorst E, Koeter M, van der Gaag M, Nieman DH, Fett AKJ, Smit F, et al. Adapted cognitive–behavioural therapy required for targeting negative symptoms in schizophrenia: meta-analysis and meta-regression. Psychol Med. 2015;45(3):453–65. 10.1017/S0033291714001147. 24993642

[63] Sitko K, Bewick BM, Owens D, Masterson C. Meta-analysis and meta-regression of cognitive behavioral therapy for psychosis (CBTp) across time: the effectiveness of CBTp has improved for delusions. Schizophrenia Bull Open. 2020;1(1). 10.1093/schizbullopen/sgaa023.

[64] Scheffler RM, Arnold DR. Projecting shortages and surpluses of doctors and nurses in the OECD: what looms ahead. Health Econ Policy Law. 2019;14(2):274–90. 10.1017/S174413311700055X. 29357954

[65] Garety PA, Fowler D, Kuipers E. Cognitive-behavioral therapy for medication-resistant symptoms. Schizophr Bull. 2000;26(1):73–86. 10.1093/oxfordjournals.schbul.a033447. 10755670

[66] O’Keeffe J, Conway R, McGuire B. A systematic review examining factors predicting favourable outcome in cognitive behavioural interventions for psychosis. Schizophr Res. 2017;183:22–30. 10.1016/j.schres.2016.11.021. 27889383

[67] Lincoln TM, Rief W, Westermann S, Ziegler M, Kesting M-L, Heibach E, Mehl S. Who stays, who benefits? Predicting dropout and change in cognitive behaviour therapy for psychosis. Psychiatry Res. 2014;216(2):198–205. 10.1016/j.psychres.2014.02.012. 24602992

[68] Health Quality Ontario. Cognitive behavioural therapy for psychosis: a health technology assessment. Ont Health Technol Assess Ser. 2018;18(5):1. PMC6235075 30443277

[69] Parry GD, Crawford MJ, Duggan C. Latrogenic harm from psychological therapies – time to move on. Br J Psychiatry. 2016;208(3):210–2. 10.1192/bjp.bp.115.163618. 26932481

[70] Klingberg S, Herrlich J, Wiedemann G, Wölwer W, Meisner C, Engel C, et al. Adverse effects of cognitive behavioral therapy and cognitive remediation in schizophrenia: results of the treatment of negative symptoms study. J Nerv Ment Dis. 2012;200(7):569–76. 10.1097/NMD.0b013e31825bfa1d. 22759932

