# Supplementary material for: Cognitive behavioral therapy for psychosis: a cost-effectiveness study using the EPiSODe model
Source: Eur Psychiatry. 2025 Jun 30;68(1):e150. doi: 10.1192/j.eurpsy.2025.10028 (PMC12538173; doi:10.1192/j.eurpsy.2025.10028)
Supplement: Konings et al. supplementary material [file S092493382510028Xsup001.docx]

Appendix A: Glossary with Health Economic terms

**Utility Weights**

Utility weights are numerical values ranging from 0 (equivalent to death) to 1 (perfect health) that represent the quality of life associated with a specific health state. These weights are used in health economic evaluations to adjust life years for quality by multiplying time spent in a health state by the utility weight. Utility values corresponding to health states are often determined with instruments such as Time Trade-Off (TTO) methods or questionnaires such as EQ-5D or SF-36.

**Quality-Adjusted Life Year (QALY)**

A QALY is a metric used in cost-utility analysis to combine the length and quality of life into a single measure. One QALY equals one year of life in perfect health, while less than perfect health is expressed as a fraction of one QALY. QALYs are commonly estimated by multiplying life years with a utility weight.

**Discounting / Discount Rate**

Discounting adjusts future costs and health benefits to their present value, reflecting the principle that people value immediate outcomes more than future ones. The discount rate represents the percentage used to calculate these adjustments, commonly set at 3-4% per year in health economics.

**Health Economic Decision Model**

A health economic decision model is a structured framework used to simulate real-world clinical and economic outcomes of healthcare interventions. It provides a systematic approach for comparing costs and outcomes of different options, often based on inputs from clinical trials, observational data, and expert opinion. These models usually aim to describe processes using model states representing health or healthcare use states, transitions representing events, and time modeled either discretely or continuously.

**Model States**

Model states are discrete health conditions or outcomes defined within a health economic decision model. For example, in a Markov model, states might include "healthy," "disease progression," "hospitalized," or "death." Patients transition between these states over time based on predefined probabilities. These probabilities are often derived from clinical trial data, observational studies, or expert consensus and are used to capture the natural history of a disease or the impact of an intervention.

Appendix B: Sample inclusion & exclusion

Administrative data from the major SMH providers in the catchment area was available. Patients were included in the study if they had one of the following DSM-IV diagnosis during study follow-up: a primary DSM-IV 295 (schizophrenia), 291 (alcohol induced psychosis), 292 (drug induced psychosis), 293 (psychosis due to medical condition), 297 (shared psychotic & delusional disorder), or 298 (brief & not otherwise specified psychotic disorder) diagnosis. Patients were excluded if they had a registered CBTp contact during study follow-up.

Appendix C: Model parameters and uncertainty

| **Schizophrenia health state** | **Patient sample** | **Lay person sample** |
| --- | --- | --- |
| **Stable** | 0.919 (0.023) | 0.865 (0.021) |
| **Relapse with outpatient care** | 0.762* (0.042**) | 0.672* (0.033**) |
| **Relapse with inpatient care** | 0.604 (0.042) | 0.479 (0.033) |

Patient sample reflects TTO responses by patients, Lay person sample TTO responses by the general public.

*Interpolated value

**Missing SE due to interpolation, largest known uncertainty used as replacement.

**Table OS1: QoL utility weight based on Briggs et al. (45)**

| Parameters | Variation | Ranges |
| --- | --- | --- |
| Cost regression model coefficients | Normal distribution | Based on estimated SEs |
| Sojourn time distribution parameters | Lognormal distribution | Based on estimated SEs |
| Mortality distribution parameters | Lognormal distribution | Based on estimated SEs |
| CBTp rehospitalisation RR | Lognormal distribution | 0.79 [0.60, 1.04] |
| QoL weights | Beta distribution | 0.865 [0.824, 0.906]  0.672 [0.607, 0.737]  0.479 [0.414, 0.544] |

SE = Standard Error; CBTp = Coginitive Behavioural Therapy for psychosis; RR = Relative Risk;

QoL = Quality of Life.

**Table OS2: Parameters included in PSA analysis.**

Appendix D: Additional scenarios

Table OS4 shows the mean results for the sensitivity analysis using varying treatment costs. Higher treatment costs decrease the cost gains, and do not affect the expected health differences.

| Discount rate | Expected cost diff p.p. | Expected QoL diff p.p. | ICER |
| --- | --- | --- | --- |
| 16 Sessions, €108.22 per hour (base case) | €492 | 0.038 years | €12,947 |
| 16 Sessions, €113.28 per hour | €557 | 0.038 years | €14,658 |
| 16 Sessions, €130.23 per hour | €774 | 0.038 years | €20,368 |
| 16 Sessions, €140.12 per hour | €900 | 0.038 years | €23,684 |
| 20 Sessions, €108.22 per hour | €838 | 0.038 years | €22,053 |
| 20 Sessions, €113.28 per hour | €919 | 0.038 years | €24,184 |
| 20 Sessions, €130.23 per hour | €1191 | 0.038 years | €31,342 |
| 20 Sessions, €140.12 per hour | €1349 | 0.038 years | €35,500 |

**Table OS4: Overview of expected cost and QoL differences per person resulting from CBTp treatment, sensitivity analysis with varying treatment costs.**

Appendix E: Stakeholders and patient engagement

The current study and model development were performed within the context of the IMPROVE project, which is a collaboration between the Rob Giel Research center (consisting of six large mental health care organizations), UMCG Psychiatry department, HTA-unit of the UMCG, Netherlands Institute of Mental Health and Addiction (Trimbos-institute), MIND Landelijk platform Psychische Gezondheid (MIND), an umbrella organization in mental health and “De Friesland Zorgverzekeraar”, a large regional health insurer. The IMPROVE Project is funded by Stiching De Friesland (Grant number DS29).

As various stakeholders were included in this project, the current study and model development were repeatedly discussed at various meetings with the relevant parties. The relevant parties had no objections to the current study and model. Some language and terminology used in this paper was adjusted following internal discussions with the relevant stakeholders.
